# Chiropractors’ views on the use of patient-reported outcome measures in clinical practice: a qualitative study

**DOI:** 10.1186/s12998-018-0219-6

**Published:** 2018-12-18

**Authors:** Michelle M. Holmes, Felicity L. Bishop, David Newell, Jonathan Field, George Lewith

**Affiliations:** 1 Department of Psychology, University of Southampton, Southampton, SO17 1BJ UK; 2 AECC University College, Bournemouth, BH7 2DF UK; 3 Back2Health, Portsmouth, Southsea PO4 0DW UK; 4 Primary Care and Population Science, University of Southampton, Southampton, UK

**Keywords:** Patient-reported outcome measures, Clinical practice, Implementation, Qualitative research, Musculoskeletal care

## Abstract

**Background:**

Patient-reported outcome measures (PROMs) are widely available for use in musculoskeletal care. However, there is little research exploring the implementation of PROMs in clinical practice. This qualitative study explored chiropractors’ views on PROMs to identify any barriers and facilitators to implementing PROMs in chiropractic care and the training needs of chiropractors regarding the use of PROMs.

**Methods:**

A qualitative study of chiropractors’ views on PROMs was undertaken as part of a larger project to address the feasibility of conducting a randomised controlled trial of PROM use in chiropractic clinics for patients with low back pain. Contact was made with chiropractors working in chiropractic companies with multiple clinic sites**.** Semi-structured interviews were conducted with eight chiropractors, either face-to-face at their place of work or over the telephone. The interviews were transcribed verbatim and analysed using thematic analysis. The data were coded inductively by two authors.

**Results:**

Chiropractors discussed their knowledge and engagement with PROMs in clinical practice, identifying reasons for their use, such as understanding clinic performance, clinical practice, and research. They also discussed how they used PROMs within their clinical practice and the benefits of using them with individual patients, for example during the consultation, identifying yellow flags, and tracking patient progress. Chiropractors voiced concerns about patient engagement with PROMs, questioning if patients find them burdensome, and the appropriate PROMs to use with patients with pain. Finally, chiropractors acknowledged the organisational barriers and facilitators to using PROMs within their practice, such as busy practices, electronic systems, and use of reception staff.

**Conclusions:**

Using participating chiropractors’ views of PROMs, the study identified barriers and facilitators to implementing PROMs in chiropractic care, such as clinician knowledge, engagement, and organisational concerns and identified the potential training needs of chiropractors regarding PROMs. The results from the study suggested chiropractors use PROMs with their individual patients, but PROMs should be meaningful to patients and chiropractors to improve engagement.

## Background

Patient-reported outcome measures (PROMs) are instruments and questionnaires which can be used to collect patients’ perceptions and views on their own health [1–4]. Data are provided exclusively by the patients, typically through paper questionnaires, electronic devices, or interviews, and amount to a numerical score [5–7]. PROMs were developed for their use in randomised-controlled trials to provide a comprehensive assessment of patient experience of illness and treatment [8–10]. The use of PROMs has since been incorporated into clinical practice to evaluate health care, increase knowledge of patients’ disease trajectories, and evaluate the effectiveness of treatments [8]. Their use is increasingly recommended in day to day clinical practice [11].

Since their development, research has been conducted to identify the impact of PROMs when used in clinical practice. Literature indicates that PROMs may impact clinically and psychologically on patients when used in clinical practice. One review of PROM research suggested that using PROMs may influence detection of psychological problems and facilitate communication between healthcare professionals and patients [12]. Three reviews, examining evidence from RCTs or controlled trials, identified that PROMs may improve the process and outcome of care [1, 13, 14]. This included improvements in health status and functional status as well as increased diagnosis and use of health services, with improvements in patient-clinician communication and patient satisfaction.

Although PROMs are increasingly being used to collect patient outcomes, with a NHS report in 2008 highlighting the importance of using PROMs to measure patients’ perspective on their care [5], there is currently little research on the implementation of PROMs in clinical practice. For PROMs to be correctly and appropriately utilised in clinical practice it is essential to understand the implementation.

Despite the availability of musculoskeletal PROMs, there has been very little published research in the context of musculoskeletal care and PROMs, with just three studies exploring how PROMs can be used in clinical practice [15–17]. Much of the literature examining the barriers and facilitators to successful PROM implementation has focused on oncology, palliative care, or mental health settings. One systematic review examined issues of implementation with allied health professionals [18]. These authors identified four potential factors affecting implementation: clinician knowledge and perceived value of PROMs, organisational support, practical barriers, and clinician concerns and consideration of patient benefit. A recent feasibility study identified that implementing a web-based PROM for a UK cohort of chiropractic practitioners was achievable and could generate information useful for patients, clinicians, and policy makers [19].

Chiropractors are increasingly encouraged to use PROMs within their practice [20], however it is currently unknown how many chiropractors do so. A survey of 62 chiropractors found that although visual analogue scales and numeric rating scales are commonly used, chiropractors only occasionally use condition-specific PROMs (such as the Oswestry Questionnaire) and generalised PROMs (such as the SF-36) [21]. Bussières et al. [22] suggest that effort should be placed on addressing this knowledge-to-practice gap and improve implementation of PROMs in clinical practice.

It is necessary to further explore the barriers and facilitators of using PROMs within the specific context of chiropractic care to ensure successful implementation. It is important to understand patients’ and clinicians’ views on using PROMs and the values of implementation. Greenhalgh et al., [11] identified that further research is required to examine how PROMs are supported in different healthcare professions, the needs of healthcare professionals aiding their use of PROMs in clinical practice, and the place of training on utilising PROMs data. This article describes a qualitative study of chiropractors’ views on PROMs, undertaken as part of a larger project to address the feasibility of conducting a randomised controlled trial of PROM use in chiropractic clinics for patients with low back pain. The findings from this study will inform a future randomised controlled trial. The aim of this qualitative study was to identify any barriers and facilitators to implementing PROMs in chiropractic care and the training needs of chiropractors regarding the use of PROMs.

## Methods

### Participants

A qualitative study was conducted interviewing chiropractors to explore their views on using PROMs in clinical practice. Participants were recruited within the South of England using convenience sampling. The South of England is home to over 1.7 million people, with over 1 million people of working age. Over 3 months, three different chiropractic services with multiple clinics (1–5) were approached. These were identified by the study team through conversation via professional networks and groups, but not by MMH, as clinics currently using PROMs. The practices are located in residential areas of large towns (population of 20,000-100,000) and smaller market towns (population of 1000-20,000). Recruitment took place simultaneously with transcription and analysis. Over the 3 month recruitment period, all chiropractors working within these practices were invited for interview (*n* = 40). Chiropractors received an email invitation to the study, explaining the purpose of the study and the procedures involved in participating. Recruitment continued until data saturation was reached (no new themes were generated from the data), this was assessed by two authors. This study received ethical approval and research governance approval from the University of Southampton (ID: 20715). All participants provided informed consent to participate in the interviews. Additionally, the data collected fit with the Data Protection Act of 1988 and the Data Policy of the University of Southampton [23, 24].

### Data collection and analysis

This research adopted a pragmatist philosophy and approach, using a qualitative methodology to allow for exploration into stakeholders subjective evaluations of using PROMs in clinical practice [25, 26]. Semi-structured interviews were conducted with participants following an interview guide (see Table 1). Interviews allow for participants to express themselves, giving individuals a chance to tell the story of their experiences [27, 28]. Interviews were conducted in private, either face-to-face at their place of work or over the telephone. The telephone interviews did not differ in content or length to those undertaken face-to-face. The interviews were undertaken by the lead author, who has previous experience in qualitative projects, as part of her PhD project. MMH is not a chiropractor but has clinical experience (previously worked for 5 years as a massage therapist), with no previous relationship to the participants. The research topic and how the study had arisen was explained to participants, and that the researcher was independent of their clinic and employers. Interviews were audio recorded and field notes were made during and immediately after the interview.

**Table 1:** Interview topics

Can you tell me about your experiences of using health questionnaires in your practice?	
*Prompts – use of data, frequency of use, decisions, discussions about data*	
What are your views on the collection and use of these questionnaires?	
*Prompts – benefits, barriers*	
How do you feel about the routine collection of this data being used in your clinical practice?	
*Prompts – feasibility, facilitating use, training*	
Literature suggests that collecting this data and providing it to practitioners may impact on patient care, how do you think this happens in practice?	
*Prompts – changes in practice*	

The interviews were transcribed verbatim and input into the computer-assisted qualitative software NVivo (version 10) for analysis [29]. The data were analysed using thematic analysis following the steps set out in Braun and Clarke [30]: 1) the data were coded inductively, 2) codes were examined for patterns and refined, 3) relationships and refined patterns between codes were identified and themes were developed, and 4) themes were described with representative data to support the theme. This allowed for a thorough exploration and detailed description of chiropractors’ experiences and views of using PROMs [30, 31]. The initial coding was conducted by one author [MMH], with refinement and grouping into higher-level categories by discussion of coding between two authors [MMH and FLB]. Quotes have been selected to best describe the findings, with pseudonyms given to participants. The study has been written up according to the consolidated criteria for reporting qualitative studies [32].

## Results

Eight chiropractors participated in an interview (4 males, 4 female). Chiropractors were based at four different clinics (chiropractors per clinic: 1,2,2,3), however many worked in more than one clinic within one chiropractic service or had their own separate private practice. For the interviews, 7 were completed face-to-face and 1 via telephone. The interviews had an average time of 31 min.

The interviews aimed to explore chiropractors’ views on the use of PROMs in clinical practice. This aimed to identify any barriers and facilitators to implementing PROMs in chiropractic care and the training needs of chiropractors regarding the use of PROMs. The analysis identified five themes. The following sections describe each theme with example quotes from participants.

### Clinician knowledge and engagement with PROMs

Chiropractors discussed clinician knowledge and engagement with PROMs. All chiropractors had used PROMs at some point during their practice. Half of the chiropractors used PROMs routinely within their practice, with four rarely using PROMs. Use of PROMs was varied amongst the chiropractors, some used electronic systems where PROMs were completed by patients before their clinical appointment, with others using a paper system filled in by patients at each visit. PROMs specifically mentioned included the Bournemouth Questionnaire [33], Patient Specific Functional Scale [34], Roland-Morris Questionnaire [35] and the Disabilities of the Arm, Shoulder and Hand (DASH) Outcome Questionnaire [36]. Choice of PROMs and process was also influenced by their clinic, for example the PROMs procedures within the chiropractic company they were employed at differed from their practices as an individual practitioner.

Most of the chiropractors were positive about using PROMs. They stated many reasons and benefits for using PROMs in their practice. Many chiropractors spoke about using the data for audits and for feedback: to understand personal and clinic performance, to compare practitioners and practices, and to improve practice. The data were thought to be useful to understand overall progress and satisfaction for groups of patients. “*obviously it helps you to see how your doing, as well, overall, across lots of different patients. And.. yeah sort of how your clinic is performing so it’s just making sure the patients are happy with other aspects of their care.*” – Gemma. Chiropractors also thought the data were necessary to collect for research purposes, to legitimise their practice and was important for the profession. However, one chiropractor cautioned that valid reasons to collect data are required:

*“what you don’t want to be doing is just gathering loads of data.. so you can say how wonderful you are. There is an element of that.. so as a profession we can say how wonderful we are. And we are doing for it ourselves, we aren’t really doing it for the patients in that case”* – Matthew.

Chiropractors were concerned over patient engagement, some believed that the data were only meaningful if all patients completed the questionnaires, and that they would need to significantly improve completion rates to be able to benefit from collecting data. Some chiropractors stated that many patients wouldnot report feeling dissatisfied and as a result were concerned that the data they received were positively-skewed. One chiropractor thought that those who are satisfied are more likely to complete PROMs and report a positive result. Chiropractors suggested that patients might not want to offend their chiropractor, or disappoint them, and therefore report improvement.

*“My gut feeling is that people that don’t complete are the people that aren’t happy with what you’ve done. So it’s immediately biased.”* – Cameron.

There were mixed responses to using PROMs within every day clinical practice. Some chiropractors thought it was beneficial to them, progressing as a chiropractor and improving their practice. Some chiropractors used PROMs as a tool, in combination with discussion and physical examination, to make a clinical decision: “*I have looked at data and then changed my practice, possibly because I was thinking that.. ‘that patient is not getting any better, what is happening?’ err.. looked at the data to see if that could help me at all, tried a completely different approach with the patient*” – Clare. Others did not use it within their practice, preferring to ask their patients personalised questions within the clinic to build rapport with patients: *“I’m too old to be that interested in learning new things from questionnaires, I’ve had a lot of experience in practice, and if I want to learn something it won’t be by using questionnaires.”* – Matthew.

Some chiropractors lacked clarity on the details of their clinic’s procedure relating to PROMs, especially when done via an electronic system. For example, there was uncertainty on the timing of follow-up PROMs: *“I think they get it at three months, or do they get it at four weeks. I think they get it at four weeks”* – Cameron. Others did not know whether the PROMs they used had been validated or empirically tested and therefore appropriate for use in practice. Others were unclear over what populations the outcomes were validated. PROMs are inherently subjective, there-in lies their value, however many chiropractors perceived this subjectivity as a weakness, voicing concerns that PROMs are open to interpretation and questioning the value of the data in clinical practice.

*“It’s even more subjective and open to interpretation. I mean the BQ* [Bournemouth Questionnaire] *and getting them to grade things on 1 - 10 is fairly subjective anyway. When you say “how do you feel, are you very much improved or just much improved?” You know you can go to a smiley face system can’t you.”* - James.

Participants believed that, in general, chiropractors might not have any previous knowledge on PROMs. In the interviews, chiropractors were asked about training on PROMs. Training on PROMs delivered via the internet, with e-resources and a physical guide was seen as acceptable by chiropractors: *“I do think that many practices can benefit*” – Clare. They suggested highlighting the benefits of using PROMs and the simplicity of the process. “*So assuming everybody knows absolutely nothing about them and saying ‘this is what they are, this is why they are useful, this is how it can help you’ erm.. is probably the way to go*.” – James.

### Use of PROMs for individual patients

Chiropractors discussed their use of PROMs throughout the treatment process with patients. PROMs were used from the first consultation to look at the patients’ story. They identified that sometimes the information they received from PROMs differed from patients reporting during the initial visit. One chiropractor stated PROMs enabled chiropractors to see things from the patient’s perspective.

*“I do think.. the longer you’re out in practice, either the more you pick up on things and use it, or the more settled to your routines you become and maybe forget the importance about the psychological aspect of the care, because you get so.. so sort of swaddled in your own routine, that you forget about renewing yourself. I think this sort of feedback is useful for renewing us, if we choose to use it actively.”* - Clare.

Chiropractors also talked about being able to identify ‘yellow flags’ by using PROMs. This triggered chiropractors to reassure the patient, educate them about recovery, and discuss self-management. One chiropractor stated that PROMs affected the management plans of his patients: *“For me it’s about identifying those that are at risk of not.. not responding as you would expect them to and then being able to intervene a lot earlier”* - James.

Chiropractors also used PROMs at follow up to track patients and identify any improvements. They were also used to identify patients who were not progressing. One chiropractor discussed how they used the PROMs to follow up a patient: *“I think they’d put that it hadn’t improved. So I phoned and err.. yeah we had a discussion about it, and they just started coming back”* - Charlotte. Chiropractors also mentioned discussing PROMs with patients and visually showing them their progress.

*“I did do a guy this morning, literally like that.. cos he was chronic and erm.. he’d had pain for three years, and I started seeing him about six months ago, and he came in for a sort of final check-up, cos I think there’s an argument for doing check-ups on people with chronic problems, and he hadn’t been for three months, and he’s pretty good, he’s almost fine really. And I pinged up his little graph and said ‘look, there you are’ and he went ‘oh, yeah, that’s loads better isn’t it’ so for him, it was really neat.”* – Cameron.

This visual depiction, and showing patients their improvement after a change in behaviour, was seen as positive reinforcement “*cos they can physically.. go.. ‘oh last time I remember the last time I filled that in I was 8 and now it’s only a 4’” –* James. Chiropractors also believed that PROMs improved patient adherence for treatment and self-care exercises “*for patient compliance.. if a patient is.. that is the advantage if the patient sees or remembers what they’ve done, then they themselves can see improvement*” - James. Chiropractors also thought that PROMs may improve the patient-clinician relationship.

*“So for the majority of patients I feel like it makes them, feel like we are really interested, like we want to know everything. Especially if they can fill it out at home, and then when they come in for the initial like consultation, I’ve already looked at it, and have some insight into their pain. I think it’s really good, and I think they feel.. kind of reassured by it.”* - Gemma.

Chiropractors also identified using PROMs to change treatment for their patients: *“I know I’ve ended care for patients because it hasn’t seemed to be helping, but I’ve continued care but in a different way. Erm.. because maybe it was maybe more appropriate to.. to refer to someone else”* - Clare.

Despite chiropractors identifying how they might discuss PROMs, many chiropractors reported not discussing PROMs with their patients. This was often in cases where patients were improving. Practitioners would also not talk to patients about anxiety and depression unless they scored highly. Several chiropractors thought discussing PROMs may only be beneficial for certain patients, such as with chronic patients. *“I suppose it gives me a handle on the psychosocial stuff and the barriers, and the chronic ones. And therefore I might tweak them more psychosocially, or more in terms of exercise and something different, from just treating.*” – Cameron. One chiropractor also said discussion was down to their personal rapport with the patient:*“So for example, if I’ve got a builder in, who’s talking about football, or rugby, I’m not going to ask him.. I’m not going to start talk about emotions and stuff like that”* - Neil.

### Patient engagement with PROMs

Chiropractors voiced views on patient engagement with PROMs. Charlotte felt that for patients: “*This is just a pain, literally*”; she along with other chiropractors expressed that PROMs were bothersome for patients, stating that patients do not enjoy completing a large volume of paperwork. Some chiropractors reported comments from patients, asking them to stop sending the questionnaires, although one chiropractor did state this was a small minority of patients. Chiropractors also had concerns that it might put off patients coming back to the practice, due to the paperwork involved *“I mean the more questionnaires you give people, the more annoyed they are going to be with questionnaires*” – Cameron.

Participants did acknowledge some barriers to patients completing PROMs, such as: email and computer access, IT skills, literacy, age, and time. Chiropractors stated that some patients might be too busy to fill them in. Some chiropractors believed that you cannot change patient engagement with PROMs *“there’s a small minority that kind of just can’t be bothered to do it, and are just not interested”* - Gemma. However, many participants had ideas to improve patient engagement. Chiropractors explained that most patients come in for treatment and are not necessarily expecting to fill out a form, and they do not understand why they are filling in the questions. One method recommended to improve engagement, was to explain to patients that completing the questionnaires is a component of their care and explain its inherent value. For example: *“this a positive thing as part of your management plan, which gives us information about how you are improving*” – James. Chiropractors also suggested reception staff may have to explain this to patients, as they often explain and provide the questionnaires to patients in advance of their appointment with the chiropractor.

*“I think it totally hinges on how you explain it to the patient and the way that they.. that initial conversation and how it’s explained initially will probably determine whether that patient stays with that or not. The better it’s explained and the more they understand it, the better their compliance will be later down the line.”* - Rachel.

### Types of PROM constructs

Chiropractors discussed some of the PROMs currently used in clinical practice for low back pain. Chiropractors also expressed wanting to focus more on a functional scale rather than a pain or quality-of-life scale. Although chiropractors acknowledged the need to know how much pain patients are in, several expressed concerns about getting patients to routinely quantify their pain. One chiropractor expressed that this might remind patients of their pain. *“I always think ‘we keep going back to the pain’ and really we don’t want them to focus on the pain.”* - Gemma. Several chiropractors commented that these pain scores might have a negative effect for the patient.

*“By always focusing on how much things hurt, and how difficult it is to do things, and how bad it’s been, for that kind of patient.. you’re perhaps, kind of, maintaining them in that, sort of, slightly negative spiral.”* - Rachel.

Chiropractors often chose to focus on functional outcomes, changing the focus to a patient’s abilities rather than their pain.

*“I work both here and in a private practice, tried different things in both places, and really ended up with.. the same thing overall.. going with measures of what they feel they can achieve and do. In my opinion I feel that’s what matters in the end.”* – Clare.

Some chiropractors also had concerns about questionnaires that used the word ‘depressed’. Noting that although this was pertinent for some patients, this may not always be appropriate.

*“It says something about.. yeah.. ‘how depressed have you been feeling?’ and she was like ‘I didn’t think I was supposed to be depressed with back pain’. And it hadn’t even crossed her mind”* - Rachel.

### Organisational barriers and facilitators

Chiropractors discussed the organisational barriers and facilitators to implementing PROMs within routine clinical practice. Chiropractors also voiced concerns about practicalities and human error. Chiropractors often spoke about forgetting to look at the data and that PROMs did not come to mind easily. It was an active task to remember to look at PROMs during follow-up treatments. They also discussed their busy practices and that time was an important factor. Chiropractors expressed that in practice they are trying to be efficient, on time, so they do not keep the patients waiting, and this can restrict their use of PROMs within treatment sessions.

“*I do think time pressure, is a big limitation, forgetting and then sometimes you think.. ‘oh I forgot on that one patient, or no I’m just going to leave that one patient out, I need to get on to the next one’ - that’s the challenge*.” - Clare.

One debate was the best medium to use for PROM collection, to use paper or via an electronic system. One chiropractor highlighted that results were easier to view and use when on the computer. The computer system they used generated patients’ results into a graph, which would be time consuming to do if PROMS were paper-based. However, some chiropractors had difficulties with using the electronic system *“You can sit there for ages trying to figure out”* - Rachel. One chiropractor had issues with the electronic system not functioning correctly. However, using a paper version often required more administrative time for both the reception staff and the chiropractor. One key message was that all data should be on the same medium. One practice used paper clinic notes and an electronic system for PROMs, which was deemed to be inconvenient. Chiropractors within this clinic spoke about the difficulties switching between the two systems when the clinic is busy, and PROMs often got forgotten.

One key facilitator in the collection of PROMs was reception staff. All chiropractors spoke very highly of their reception teams. Some chiropractors noted it was a joint effort, with reception staff explaining the importance of PROMs to patients and chasing patients for follow-ups. One chiropractor, who did not have the support of a reception team, noted that this would improve data collection. However, other chiropractors saw PROMs as mainly an administrative task that they did not get involved with: “*I just leave it to the receptionist to do all that*” - Cameron.

## Discussion

This study aimed to explore chiropractors’ views on the use of PROMs in clinical practice. In order to address the knowledge-to-practice gap of using PROMs, it is essential to understand the implementation process [22]. Five themes relating to the chiropractors’ use of PROMs were developed during the analysis. The findings of the qualitative interviews and existing literature should be considered for chiropractic practices contemplating implementing PROMs into their clinical practice. Antunes et al. [37] previously conducted a review of barriers and facilitators of implementing PROMs in the palliative care setting. The review suggested a series of steps that need to be taken prior to implementing PROMs in clinical practice, this included: selection of outcome measure, decision of application of measure, and clinician education. The findings of this study have identified a series of barriers and facilitators to implementing PROMs in chiropractic care and the training needs of chiropractors regarding the use of PROMs.

### Chiropractor preference and selection of PROMs

Within the interviews, chiropractors stated a preference for functional scales, which focus on a patient’s functioning rather than pain levels, with chiropractors finding them meaningful for their clinical practice. These preferences will need to be addressed, both in selection and practitioner education on appropriate measures, for PROMs to be successful in clinical practice. One systematic review has similarly identified that PROMs must be appropriate for successful implementation and use in clinical practice [18], suggesting PROMs must be clinically meaningful for chiropractors to engage with them. Clinicians in mental health settings also suggested that PROMs must be of clinical value in order for clinician engagement [38].

### Application of PROMs

Although two chiropractors suggested issues with an electronic PROM system this was preferred over a paper-based system. In a study to assess the feasibility and acceptability of PROMs with patients with rheumatoid arthritis, patients’ perceived a web-based PROM as easy, and reported willingness to fill in the questionnaires at home [39]. During the feasibility study interviews, IT skills, literacy, age, and time were suggested as barriers to completion. However, as these are chiropractors’ perceptions on barriers, further research is needed to explore patients’ views on completion of PROMs.

A commentary by Chang [40] also identified that unsuitable or poorly designed electronic PROM software is a barrier to successful implementation. Some chiropractors felt that PROMs were an administrative task and so it is necessary to ensure both a conducive environment and improve knowledge of the use of PROMs to improve engagement. This could be addressed by ensuring computerised PROMs are designed to be easily used in busy practice settings and providing training on PROMs to clinicians.

Interviews also explored how to improve patient engagement. Two reviews previously identified that co-operative colleagues and staff encouragement facilitated patient engagement and the use of PROMs in clinical practice [18, 37]. Chiropractors suggested that reception staff could explain to patients that PROMs are a valuable part of their care. This may increase fidelity to PROMs in clinical practice.

### Clinician education and implementation

The qualitative interviews identified that chiropractors often had a lack of knowledge and engagement with PROMs, despite using them in clinical practice. It is also acknowledged in the findings that chiropractors’ attitudes and beliefs around the consultation process may influence their use of PROMs, with some chiropractors explicitly rejecting the use of PROMs, despite clinical procedures. Antunes et al. [37] and Duncan and Murray [18] also identified clinicians’ lack of knowledge and education was a significant barrier to PROM use. Literature suggests that educating clinicians on the purposes of PROMs and the benefits of using them may be beneficial [18, 37, 38]. An additional barrier may be chiropractors’ confidence in managing the issues identified by using PROMs. A systematic review found that whilst physiotherapists feel confident with the mechanical treatment of low back pain, many feel unprepared to treat the cognitive, social, and psychological factors that contribute to recovery [41]. Therefore, training should include both how to identify these factors using PROMs and how to manage these factors within their clinical domain.

Chiropractors indicated that training would be acceptable and would improve knowledge and engagement with PROMs in clinical practice. It is important that training should also include the use of PROMs for clinical audit, feedback, and research purposes. Understanding the multitude of ways PROMs can be used within clinical practice may improve uptake and engagement.

### Methodological implications

Generally, the results suggest that clinician training, selection of PROMs, and organisational barriers should be considered when implementing PROMs in clinical practice. However, the sampling of participants may limit the transferability of the results. Socioeconomical region of the clinics and details on the experience and training of the sample were not collected. It is therefore not possible to compare the chiropractors to the wider profession. The findings also only represent the views of chiropractors who have volunteered to take part in research and therefore may not be representative of the population. These findings also do not include the patient perspective of PROMs. While the sampling may limit the transferability, this study provides an insight into PROMs use in chiropractic practice.

### Recommendations for research

Further research in this area should consider the views of patients completing PROMs in chiropractic practice. Patients’ views will provide necessary insight into the process and benefits of implementing PROMs into clinical practice. Additionally, as interviewees spoke about how they use PROMs with individual patients, research should also explore how PROMs may potentially affect the process and outcomes of patient care when used clinical practice. A recent review of PROMs examining their use with individual patients found PROMs could influence the clinician-patient relationship, consultation discussions, and decision-making around treatment [11]. Not all patient populations benefit from PROMs completion, with a review of PROMs in psychological care for those with common mental disorders, finding no difference in treatment outcome and limited evidence in improving patient satisfaction [42]. Further research is required to understand which healthcare services PROMs should be incorporated into and how this may impact patients. The findings from this study will inform a future randomised controlled trial to explore the clinical and psychosocial effects of PROM use in chiropractic care.

## Conclusion

Chiropractors are increasingly using PROMs in their clinical practice. The aim of this qualitative study was to examine the views of chiropractors on using PROMs. Exploring chiropractors’ experience of using PROMs, this study identified how clinician knowledge and engagement and organisational barriers and facilitators affect implementing PROMs in chiropractic care, such as choosing the appropriate PROMs and systems to use in their practice. Chiropractors also identified possible training needs of chiropractors regarding PROMs, with training including the process and benefits of using PROMs in clinical practice. The results from the study also demonstrated the necessity of ensuring PROMs are meaningful to patients and chiropractors. It is clear there are differing views and engagement with PROMs within clinical practice; in addition, future research must consider patients’ views on completing PROMs and how it affects the process of clinical practice and outcomes.

## Abbreviation

PROMs: patient-reported outcome measures
